# Protein Phosphatase 5 Contributes to the Overexpression of Epigenetically Regulated T-Lymphocyte Genes in Patients with Lupus

**Published:** 2016-12-30

**Authors:** D Patel, G Gorelik, B Richardson

**Affiliations:** 1 Eli Lilly and Company, San Diego, CA, USA; 2 Rheumatology Division, Department of Medicine, University of Michigan, Ann Arbor MI, USA; 3 Department of Medicine, Ann Arbor VA Medical Center, Ann Arbor MI, USA

**Keywords:** Lupus, Atherosclerosis, T cells, Signaling, Protein phosphatase 5, DNA methylation, Oxidative stress

## Abstract

**Objective:**

Lupus develops when genetically predisposed people encounter certain drugs or environmental agents causing oxidative stress such as infections and sun exposure, and then typically follows a chronic relapsing course with flares triggered by the exogenous stressors. Current evidence indicates that these environmental agents can trigger lupus flares by inhibiting the replication of DNA methylation patterns during mitosis in CD4^+^ T cells, altering the expression of genes suppressed by this mechanism that convert normal “helper” cells into auto reactive cells which promote lupus flares. How environmental stressors inhibit T cell DNA methylation though is incompletely understood. Protein phosphatase 5 (PP5) is a stress induced inhibitor of T cell ERK and JNK signaling in “senescent” CD4^+^CD28^−^ T cells, also characterized by DNA demethylation and altered expression of genes that promote atherosclerosis. We tested if PP5 is increased in CD4^+^CD28^+^ T cells by oxidative stress, if PP5 transfection causes overexpression of methylation sensitive genes in T cells, and if PP5 is overexpressed in lupus T cells.

**Results:**

PP5 was found to be overexpressed in CD4^+^CD28^+^ T cells treated with H_2_O_2_ and ONOO− and in T cells from lupus patients.

**Conclusion:**

The results indicate that PP5 increases expression of methylation sensitive T cell genes, and may contribute to the aberrant gene expression in CD4^+^CD28^+^ T cells that characterize lupus flares as well as the aberrant gene expression in CD4^+^CD28^−^ T cells that promote atherosclerosis.

## Introduction

Systemic lupus erythematosus (SLE) is a chronic relapsing autoimmune disease that primarily affects women, and requires both a genetic predisposition and an environmental exposure for onset and flares. Exogenous agents triggering lupus flares include drugs such as procainamide and hydralazine [1] and agents causing oxidative stress such as sun exposure, infections, and others [2]. Current evidence indicates that these agents contribute to lupus flares by impairing the replication of T cell DNA methylation patterns during mitosis [2]. Procainamide is a competitive inhibitor of DNA methyltransferase 1 (Dnmt1), the enzyme replicating DNA methylation patterns during mitosis, while hydralazine inhibits PKCδ, preventing Dnmt1 upregulation during mitosis [3]. Inhibiting T cell DNA methylation causes aberrant overexpression of genes that convert normal “helper” CD4+ T cells into autoreactive, inflammatory and cytotoxic cells that are sufficient to cause lupus-like autoimmunity in animal models [4].

CD4^+^ T cell DNA methylation is impaired in patients with active lupus. The genes encoding perforin (PRF1), CD11a (ITGAL), CD70 (TNFSF7), CD40L (CD40LG), and the killer cell immunoglobulin-like receptor (KIR) genes are normally suppressed by DNA methylation in CD4^+^ T cells, but are demethylated and over-expressed by CD4+ T cells from patients with active lupus [5,6]. CD11a demethylation and overexpression contributes to T cell autoreactivity [7], while CD70 and CD40L overexpression contribute to B cell overstimulation [8] and perforin to cytotoxic responses [9,10], while the aberrantly expressed KIR genes contribute to IFN-γ secretion and regulate autologous macrophage killing [11]. Notably, CD40L is encoded on the X chromosome, so T cells from men have one, expressed CD40LG gene while T cells from women have one active gene and one methylated and silenced gene. Inhibiting DNA methylation in CD4^+^ T cells from women causes CD40L overexpression while DNA methylation inhibition has no effect on CD40L expression by CD4^+^ T cells from men [8]. Similarly, CD4^+^ T cells from women with active lupus overexpress CD40L but CD4^+^ T cells from men with active lupus do not [8]. Further, mice receiving syngeneic CD4^+^ T cells in which DNA methylation has been inhibited *in vitro* [12], and mice with an inducible T cell DNA methylation defect [13] develop lupus-like autoimmunity [14]. Thus, changes in T cell gene expression secondary to DNA demethylation can contribute to flares of lupus-like autoimmunity. However, the mechanisms causing T cell DNA demethylation in lupus are not completely understood. Identifying the mechanisms responsible may lead to new ways to prevent and treat lupus flares.

Dnmt1 is the enzyme that replicates DNA methylation patterns during mitosis [1]. Resting T cells have low Dnmt1 levels, but as T cells enter mitosis this enzyme is upregulated by signals transmitted through the ERK and JNK pathways [15]. Dnmt1 levels, as well as ERK pathway signaling are decreased in CD4^+^ T cells from lupus patients [16]. Importantly, decreasing Dnmt1 levels or enzymatic activity during mitosis with enzyme inhibitors like 5-azacytidine or procainamide [12,17], signaling inhibitors like U0126 or hydralazine [17], or with siRNA’s targeting signaling molecules in the ERK or JNK pathways [18], prevents the methylation of newly synthesized CD4^+^ T cell DNA, and increases expression of genes normally suppressed by this mechanism including CD11a [19], KIR [6], perforin [9], CD40L [8] and CD70 [20].

Protein phosphatase 5 (PP5) is a stress induced protein that inhibits signaling through both the ERK and JNK pathways [21]. PP5 deactivates ASK1 to inhibit the JNK pathway and dephosphorylates Raf-1 to inhibit the ERK pathway [21–25]. PP5 levels are increased in “senescent” CD4^+^CD28^−^ T cells from the elderly and patients with chronic inflammatory diseases like rheumatoid arthritis and others. These cells infiltrate atherosclerotic plaques, promoting their growth and rupture [26]. CD4^+^CD28^−^ stress-induced, PP5 overexpressing T cells have decreased ERK and JNK pathway signaling, low Dnmt1 levels, and overexpress methylation sensitive genes including KIR2DL4, CD70 and perforin [18,27], similar to the epigenetically altered CD4^+^CD28^+^ T cells from patients with active lupus.

Epigenetic effects of PP5 overexpression have not been studied in T cells or autoimmunity. We therefore tested if PP5 is overexpressed in CD4^+^ T cells from patients with lupus and if PP5 decreases T cell Dnmt1 expression and causes overexpression of genes normally suppressed in T cells by DNA methylation. The results indicate that PP5 is a previously undescribed regulator of T cell Dnmt1 expression as well as the expression of methylation sensitive genes that contribute to lupus pathogenesis. These observations indicate a novel mechanism by which environmental stressors such as oxidative stress and others may contribute to lupus flares.

## Methods

### Subjects

Female lupus patients with inactive and active disease were recruited from the outpatient Rheumatology clinics at the University of Michigan. Lupus patients met criteria for the classification of lupus [28], and disease activity was determined using the systemic lupus erythematosus disease activity index (SLEDAI) [29].

### PP5 expression construct

A green fluorescent protein (GFP) PP5 expression construct was generated by cloning the PP5c open reading frame (ORF) construct (Origene) into the pCMV6-GFP vector (Origene) at the EcoRI and BamHI sites, immediately upstream of the GFP ORF.

### Cell culture and transfection

Peripheral blood mononuclear cells (PBMCs) were isolated from freshly drawn venous blood of healthy volunteers and lupus patients by Ficoll density gradient centrifugation, then cultured in RPMI 1640 supplemented with 10% fetal calf serum and stimulated with phytohemagglutinin (PHA) and IL-2 using previously published protocols [30]. Twenty-four hours later CD4^+^ T cells were isolated by negative selection (Dynal, Invitrogen) and treated with 20 μM ONOOas previously described [30], or transfected with vectors encoding GFP or a GFP-PP5 fusion protein by nucleofection as per the manufacturer protocols (Amaxa). The cells were then cultured then analyzed by flow cytometry as described below.

### Flow cytometry

Epigenetically altered T cells were identified with PE-anti-Kir2DL4/CD158D (clone 181703; R&D Systems, Minneapolis, MN), anti-CD40L-PE (clone hCD40L-M91), anti-CD11a-PE (clone HI111), PEanti-CD70 (clone Ki-24), and PE-Cy5-anti-CD4 (clone RPA-T4) (Becton Dickinson, Franklin Lakes, NJ). PE-anti-CD158b (clone CHL), PE-anti-CD158i (clone FES172), PE-anti-CD158b1/b2, j (clone GL183), and PE-anti-CD158a, h (clone EB6B) were from Beckman Coulter (Brea, CA) and analyzed by multicolor flow cytometry as described [31]. All antibodies were titrated to determine their optimal concentrations prior to use.

CD4^+^GFP^+^ cells were identified by gating on GFP then analyzed for KIR, perforin, CD40L, and CD70. For sorting, the cells were also stained with (4′, 6-diamidino-2-phenylindole) (DAPI), and live transfected cells (CD4^+^GFP^+^DAPI^−^) were isolated prior to analysis by RT-PCR.

### RT-PCR

Total RNA was prepared from sorted live transfected cells per manufacturer’s protocols (Qiagen) and analyzed by RT-PCR. Gene expression in GFP-PP5 transfected cells is expressed as fold change relative to cells transfected with GFP alone after normalizing with β-actin using the delta-delta-Ct (ddCt method). The primer sequences were:

β-actin forward GGACTTCGAGCAAGAGATGG,
β-actin reverse AGCACTGTGTTGGCGTACAG,
KIR 2DL4 forward AAAACTGGTATCGCCAGACACCTGC,
KIR 2DL4 reverse AGCACCAGCGATGAAGGAGAAAGA,
perforin forward caccctctgtgaaaatgccctac,
perforin reverse tccagtcgttgcggatgctac,
CD70 forward GTCACTTGGGTGGGACGTAG,
CD70 reverse GATGGATACGTAGCTGCCCC,
CD11a forward CAGTCACCCTGAGAGGTTCC,
CD11a reverse CTGGTCACACGTTCGAGACA,
Dnmt1 forward GAGCTACCACGCAGACATCA,
Dnmt1 reverse CGAGGAAGTAGAAGCGGTTG,
PP5c forward CAAGCTGAGCACGCTCGTGGAA, and
PP5c reverse CTGATCGAGCGCCCGTTCTGT.

Results are presented as the mean+SEM of 3–5 determinations/sample.

## Results

**Figure 1:**
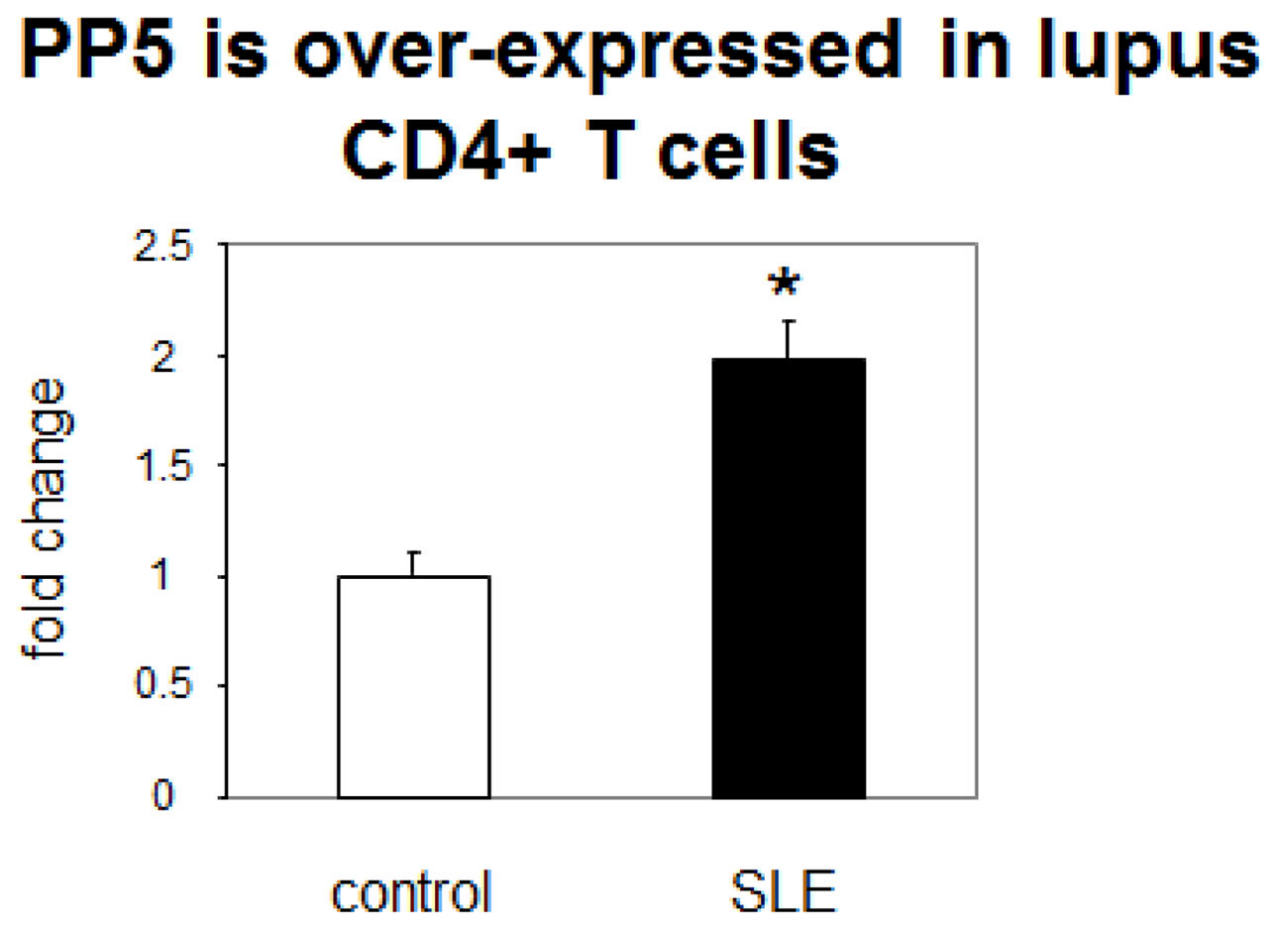
PP5 is overexpressed in CD4+ lupus T cells. PP5 mRNA levels were measured in CD4^+^CD28^+^ T cells isolated from the peripheral blood of 4 lupus patients with varying disease activity (SLEDAI=0, 2, 6, 10) and 4 age and gender matched healthy controls. *p=0.03.

PP5 expression is increased in CD4^+^CD28^+^ T cells from lupus patients. As noted above, lupus flares are triggered by environmental agents that cause oxidative stress such as infections, UV light, and others [2]. ERK pathway signaling in CD4^+^ lupus T cells is defective, due at least in part to impaired PKCδ activation [3], caused by oxidative damage [32]. PP5 expression is also increased by oxidative stress [33], and may similarly contribute to lupus flares by suppressing ERK pathway signaling, leading to decreased Dnmt1 expression and subsequent over-expression of pro-inflammatory genes. We therefore compared PP5 mRNA levels in CD4^+^CD28^+^ T cells freshly isolated from lupus patients with varying levels of disease activity to CD4^+^CD28^+^ T cells isolated from age and gender matched healthy volunteers. We found that PP5 mRNA levels are significantly higher in CD4^+^CD28^+^ T cells from lupus patients relative to controls (Figure 1). The PP5 over-expression is consistent with the decreased Dnmt1 levels and increased KIR, perforin, CD40L, CD70, and CD11a expression seen in CD4^+^CD28^+^ T cells from lupus patients [4,11]. While there was no significant relationship between PP5 levels and the SLEDAI in these subjects (P>0.05), PP5 activity is increased by interactions with other proteins such as Hsp90 [34], and Hsp90 levels increase in lupus leukocytes during flares [35].

**Figure 2:**
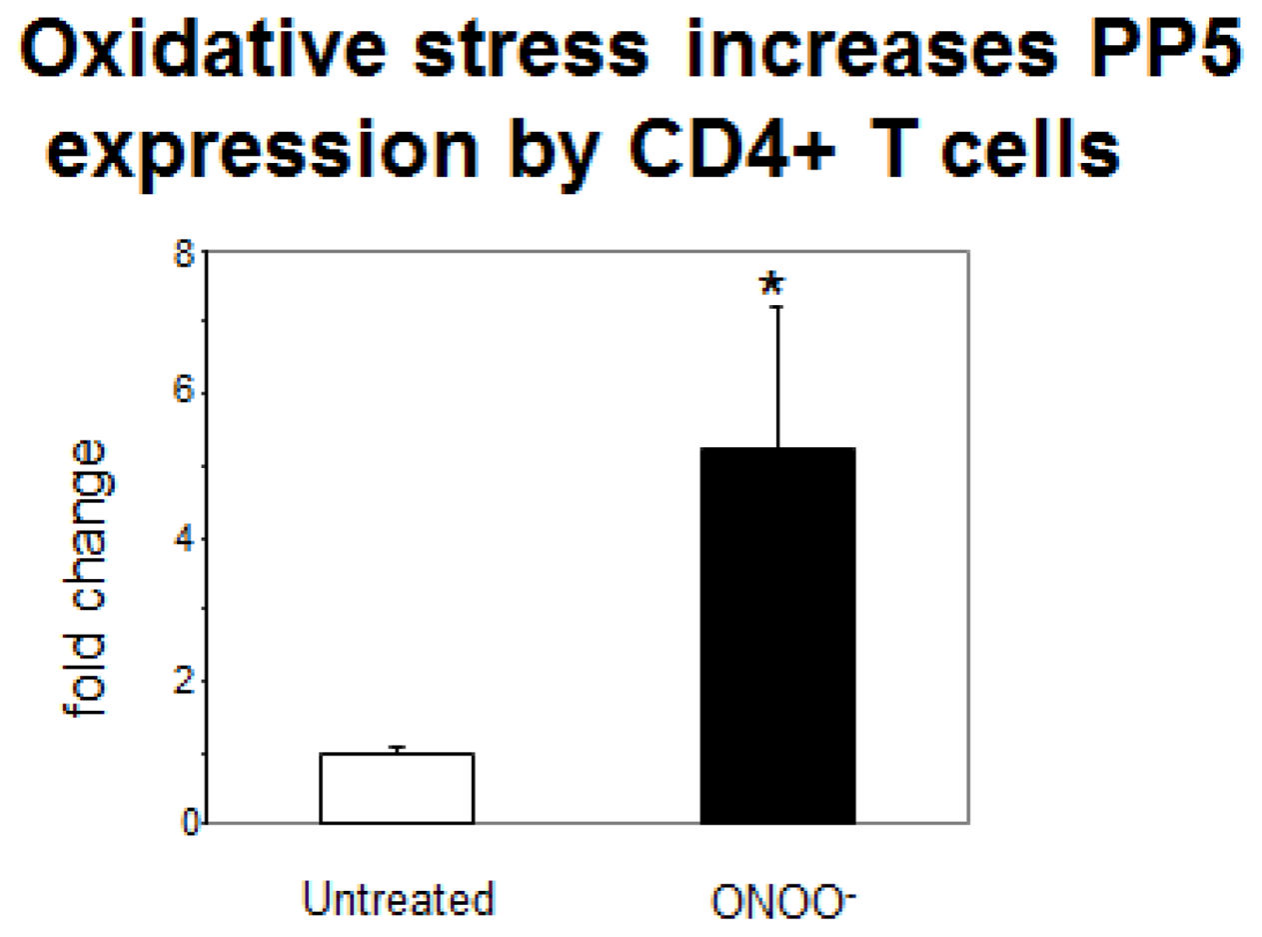
Oxidative stress increases PP5 expression in CD4^+^ T cells. CD4^+^ T cells from healthy subjects were stimulated with PHA then treated with 20 μM ONOO-. 72 hours later PP5 mRNA levels were measured by RT-PCR. N=3. *p<0.05.

Oxidative stress increases PP5 expression. As noted above, PP5 is a stress induced protein [21] and lupus flares can be triggered by agents that cause oxidative stress [4]. We have reported that treating CD4^+^ T cells with oxidizing agents causes demethylation and overexpression of methylation sensitive genes [30] and that the oxidized cells are sufficient to cause a lupus-like disease *in vivo* [36]. We therefore asked if oxidizing agents cause an increase in T cell PP5 expression. CD4^+^ T cells were stimulated with PHA then treated with 20 μM ONOO− using previously published protocols [30]. Seventy-two hours later PP5 expression was measured by RT-PCR. Figure 2 shows that ONOOincreases CD4^+^ T cell PP5 expression. We have previously reported that ONOO^−^ has a greater effect on methylation-sensitive gene expression than H_2_O_2_ [30].

**Figure 3:**
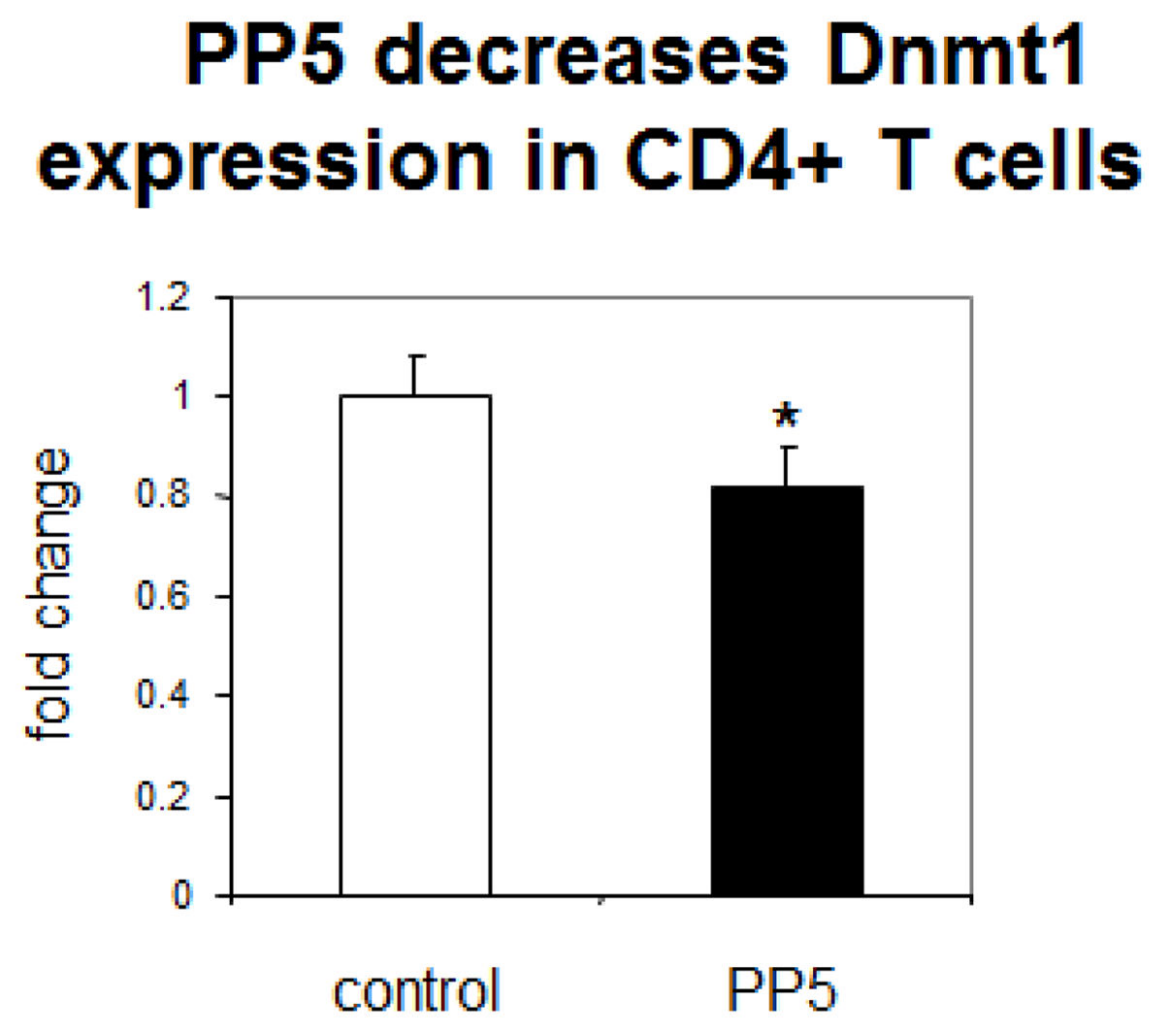
PP5 decreases Dnmt1 levels in CD4^+^ T cells. CD4^+^ T cells from healthy subjects were stimulated with PHA, transfected with a PP5-GFP expression construct and Dnmt1 mRNA levels measured 24–36 h later. N=3, *p=0.02.

PP5 decreases T cell Dnmt1 gene expression. Since PP5 decreases signaling by the ERK and JNK pathways, and these pathways are required to increase Dnmt1 expression, we hypothesized PP5 overexpression could decrease Dnmt1 gene expression. CD4^+^ T cells were transfected with the PP5 expression construct and then analyzed by RT-PCR 24–36 hours later. PP5 caused a significant decrease (20 ± 8%, n=3, p=0.05) in Dnmt1 mRNA levels (Figure 3).

We then tested if increased PP5 levels cause overexpression of the genes encoding perforin, CD11a, KIR and CD70 in CD4^+^ T cells. These genes were selected because they are over-expressed by T cells experimentally demethylated *in vitro* by treatment with Dnmt1 inhibitors and in T cells from patients with active lupus [1,11]. CD4^+^ T cells were transfected with a PP5-GFP expression construct then GFP protein expression was measured by flow cytometry. PP5 transfected CD4^+^ T cells (DAPI-GPF^+^) were sorted from GFP^−^ cells 3 days after transfection and gene expression was analyzed by RT-PCR.

**Figure 4:**
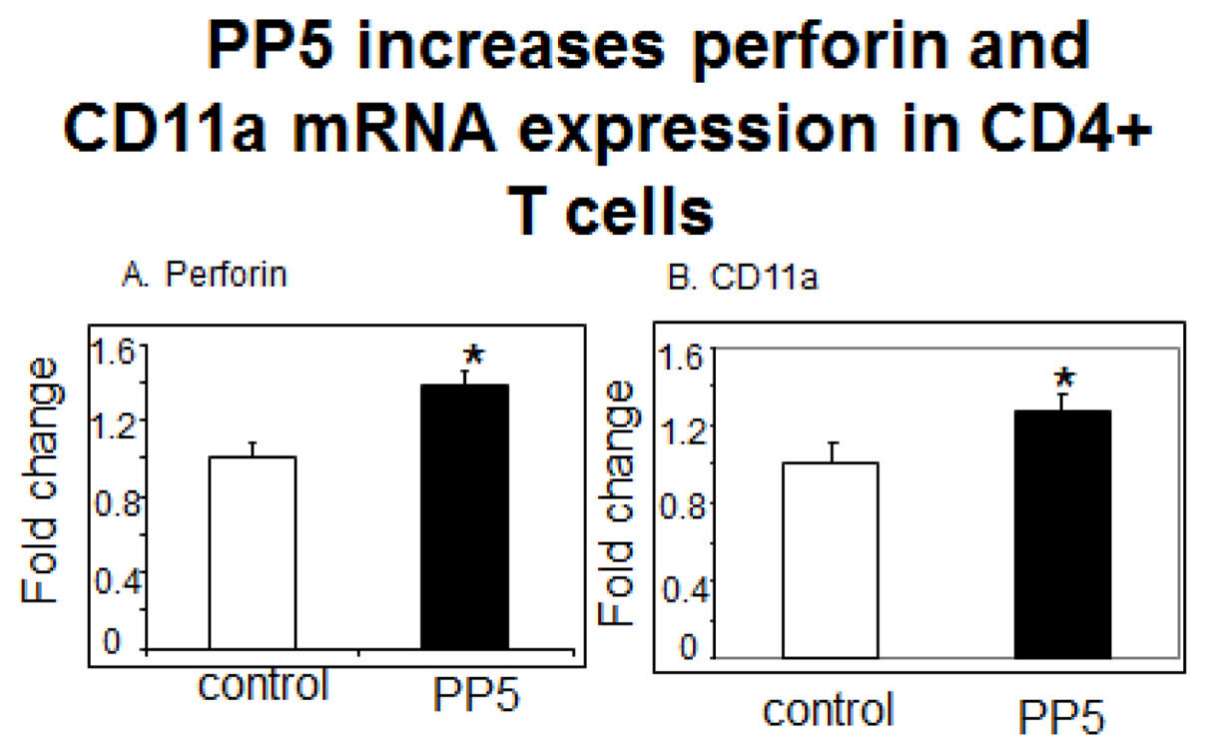
PP5 increases perforin and CD11a expression in CD4^+^ T cells. CD4^+^ T cells were stimulated with PHA, transfected with the PP5-GFP expression construct then **A**- perforin (N=4, p=0.03) and **B**- CD11a (N=5, *P=0.047) mRNA levels were measured 3 days later.

**Figure 5:**
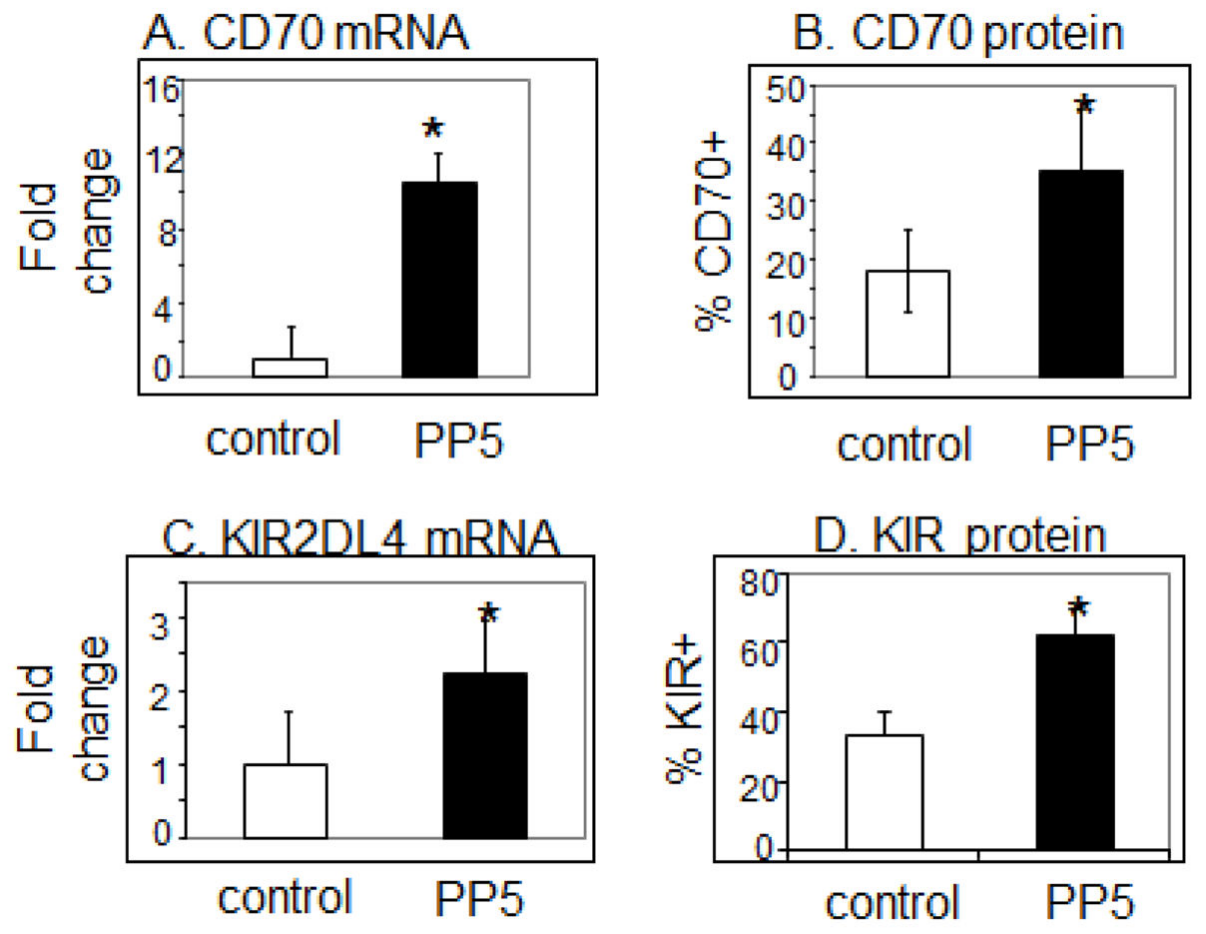
PP5 increases CD70 and KIR expression in CD4^+^ T cells. CD4^+^ T cells were stimulated with PHA, transfected with the PP5-GFP expression construct then CD70 and KIR mRNA and protein levels were measured 3 days later. **A**- CD70 mRNA levels. N=7, p=0.03. **B**- CD70 protein levels. N=3, p<0.05. **C**- KIR2DL4 mRNA levels. N=3, *p=0.04. **D**- KIR protein levels. N=7, p=0.002.

**Figure 6:**
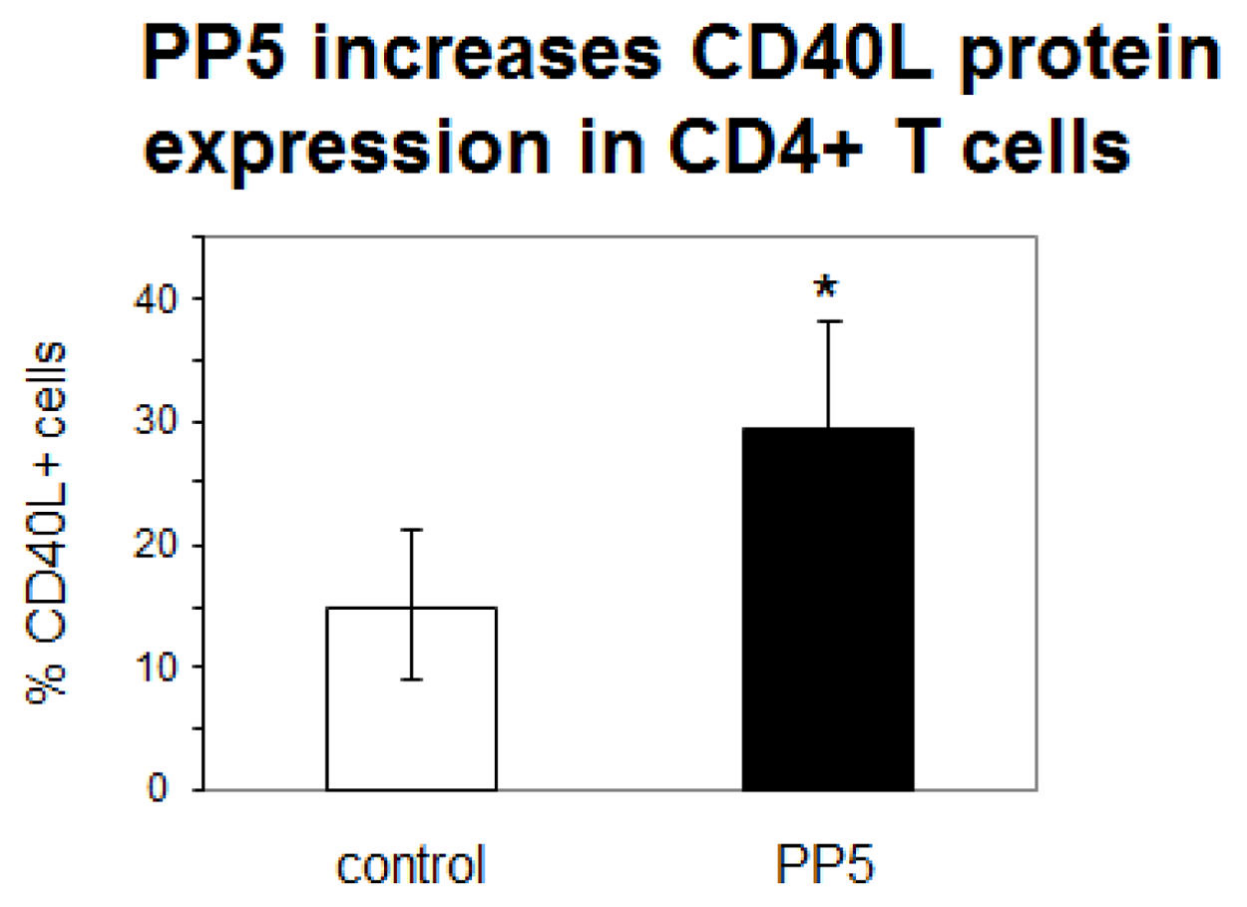
PP5 increases CD40L expression in CD4^+^ T cells. Female CD4^+^ T cells were stimulated with PHA, transfected with the PP5-GFP expression construct then CD40L surface protein was measured 3 days later. N=3, *P<0.05.

PP5 increased levels of perforin (Figure 4a, p=0.03, n=4) and CD11a mRNA (Figure 4b, p=0.047, n=5) in the CD4^+^ T cells. PP5 transfection also increased levels of CD70 mRNA (Figure 5a, p=0.03) and surface protein (Figure 5b, p<0.05), KIR2DL4 mRNA (Figure 5c, p=0.04, n=3) and KIR protein (Figure 5d, p=0.002 n=7) as well as CD40L surface protein (Figure 6, p<0.05, n=3).

Since KIR, perforin, CD70, X-linked CD40L and other genes are normally suppressed by DNA methylation in T cells [27], and Dnmt1 “knockdowns” cause demethylation and overexpression of KIR, CD70 and perforin in T cells [27], these results support the hypothesis that PP5 decreases Dnmt1 levels to activate expression of methylation sensitive genes.

## Discussion

These studies demonstrate that PP5 is a regulator of the same methylation sensitive T cell genes previously found to be overexpressed in lupus, including CD11a, perforin, CD70, the KIR genes and CD40L. PP5 negatively regulates signaling through the ERK and JNK pathways [21], and as hypothesized, Dnmt1 mRNA levels were decreased in cells transfected with PP5. PP5 has not been studied previously in autoimmunity, and has not been implicated as a regulator of gene expression in lymphocytes or other immune cells. However, microarray analysis has revealed that it is upregulated in mantle cell lymphomas, but the significance of this finding is unknown [37].

PP5 levels were recently found to be increased in CD4^+^CD28^−^ T cells generated in vitro [18]. CD4^+^CD28^−^ T cells are a unique subset found in aging and chronic inflammatory diseases [38,39]. These cells overexpress many of the pro-inflammatory and cytotoxic methylation sensitive genes including KIRs and perforin. Further, they are increased in the peripheral blood of lupus patients [11], and are implicated in the pathogenesis of atherosclerosis [40–42]. PP5 levels are also induced by oxidative stress [21], and as noted above, agents causing oxidative stress trigger of lupus flares. Further, oxidative damage impairs PKCδ phosphorylation, leading to decreased ERK pathway signaling and over-expression of pro-inflammatory genes [3,43,44]. Our results now indicate that PP5 may provide another mechanism linking oxidative stress to lupus flares by contributing to the over-expression of pro-inflammatory genes. Further, chronic stimulation of the epigenetically altered CD4^+^CD28^+^KIR^+^ T cells found in lupus patients may contribute to their conversion to senescent CD4^+^CD28^−^KIR^+^ T cells. Thus, PP5’s ability to induce proinflammatory gene expression may also contribute to the accelerated atherosclerosis seen in lupus patients [45]. A transgenic mouse that overexpresses PP5 in T cells in a doxycycline-inducible manner is currently being developed to test if PP5 causes a lupus-like phenotype and accelerated atherosclerosis in the apoE−/− model.

## Abbreviations

PP5: Protein Phosphatase 5
KIR: Killer Cell Immunoglobulin-like receptor
ORF: Open Reading Frame
GFP: Green Fluorescent Protein

## References

[1] Richardson B (2007). Primer: epigenetics of autoimmunity. Nat Clin Pract Rheumatol.

[2] Mak A, Tay SH (2014). Environmental factors, toxicants and systemic lupus erythematosus. Int J Mol Sci.

[3] Gorelik G, Fang JY, Wu A, Sawalha AH, Richardson B (2007). Impaired T cell protein kinase C delta activation decreases ERK pathway signaling in idiopathic and hydralazine-induced lupus. J Immunol.

[4] Somers EC, Richardson BC (2014). Environmental exposures, epigenetic changes and the risk of lupus. Lupus.

[5] Zhang Y, Zhao M, Sawalha AH, Richardson B, Lu Q (2013). Impaired DNA methylation and its mechanisms in CD4(+)T cells of systemic lupus erythematosus. J Autoimmun.

[6] Liu Y, Kuick R, Hanash S, Richardson B (2009). DNA methylation inhibition increases T cell KIR expression through effects on both promoter methylation and transcription factors. Clin Immunol.

[7] Lu Q, Kaplan M, Ray D, Zacharek S, Gutsch D (2002). Demethylation of ITGAL (CD11a) regulatory sequences in systemic lupus erythematosus. Arthritis Rheum.

[8] Lu Q, Wu A, Tesmer L, Ray D, Yousif N (2007). Demethylation of CD40LG on the inactive X in T cells from women with lupus. J Immunol.

[9] Lu Q, Wu A, Ray D, Deng C, Attwood J (2003). DNA methylation and chromatin structure regulate T cell perforin gene expression. J Immunol.

[10] Kaplan MJ, Lu Q, Wu A, Attwood J, Richardson B (2004). Demethylation of promoter regulatory elements contributes to perforin overexpression in CD4+ lupus T cells. J Immunol.

[11] Basu D, Liu Y, Wu A, Yarlagadda S, Gorelik GJ (2009). Stimulatory and inhibitory killer Ig-like receptor molecules are expressed and functional on lupus T cells. J Immunol.

[12] Quddus J, Johnson KJ, Gavalchin J, Amento EP, Chrisp CE (1993). Treating activated CD4+ T cells with either of two distinct DNA methyltransferase inhibitors, 5-azacytidine or procainamide, is sufficient to cause a lupus-like disease in syngeneic mice. J Clin Invest.

[13] Gorelik G, Sawalha AH, Patel D, Johnson K, Richardson B (2015). T cell PKCdelta kinase inactivation induces lupus-like autoimmunity in mice. Clin Immunol.

[14] Yung RL, Quddus J, Chrisp CE, Johnson KJ, Richardson BC (1995). Mechanism of drug-induced lupus. I. Cloned Th2 cells modified with DNA methylation inhibitors in vitro cause autoimmunity in vivo. J Immunol.

[15] Rouleau J, MacLeod AR, Szyf M (1995). Regulation of the DNA methyltransferase by the Ras-AP-1 signaling pathway. J Biol Chem.

[16] Gorelik G, Richardson B (2009). Aberrant T cell ERK pathway signaling and chromatin structure in lupus. Autoimmun Rev.

[17] Deng C, Lu Q, Zhang Z, Rao T, Attwood J (2003). Hydralazine may induce autoimmunity by inhibiting extracellular signal-regulated kinase pathway signaling. Arthritis Rheum.

[18] Chen Y, Gorelik GJ, Strickland FM, Richardson BC (2010). Decreased ERK and JNK signaling contribute to gene overexpression in “senescent” CD4+CD28− T cells through epigenetic mechanisms. J Leukoc Biol.

[19] Lu Q, Ray D, Gutsch D, Richardson B (2002). Effect of DNA methylation and chromatin structure on ITGAL expression. Blood.

[20] Lu Q, Wu A, Richardson BC (2005). Demethylation of the same promoter sequence increases CD70 expression in lupus T cells and T cells treated with lupus-inducing drugs. J Immunol.

[21] Golden T, Swingle M, Honkanen RE (2008). The role of serine/threonine protein phosphatase type 5 (PP5) in the regulation of stress-induced signaling networks and cancer. Cancer Metastasis Rev.

[22] Morita K, Saitoh M, Tobiume K, Matsuura H, Enomoto S (2001). Negative feedback regulation of ASK1 by protein phosphatase 5 (PP5) in response to oxidative stress. EMBO J.

[23] Zhou G, Golden T, Aragon IV, Honkanen RE (2004). Ser/Thr protein phosphatase 5 inactivates hypoxia-induced activation of an apoptosis signal-regulating kinase 1/MKK-4/JNK signaling cascade. J Biol Chem.

[24] von Kriegsheim A, Pitt A, Grindlay GJ, Kolch W, Dhillon AS (2006). Regulation of the Raf-MEK-ERK pathway by protein phosphatase 5. Nat Cell Biol.

[25] Takeda K, Noguchi T, Naguro I, Ichijo H (2008). Apoptosis signalregulating kinase 1 in stress and immune response. Annu Rev Pharmacol Toxicol.

[26] Dumitriu IE (2015). The life (and death) of CD4+ CD28(null) T cells in inflammatory diseases. Immunology.

[27] Liu Y, Chen Y, Richardson B (2009). Decreased DNA methyltransferase levels contribute to abnormal gene expression in “senescent” CD4(+)CD28(-) T cells. Clin Immunol.

[28] Yu C, Gershwin ME, Chang C (2014). Diagnostic criteria for systemic lupus erythematosus: a critical review. J Autoimmun.

[29] Ibanez D, Urowitz MB, Gladman DD (2003). Summarizing disease features over time: I. Adjusted mean SLEDAI derivation and application to an index of disease activity in lupus. J Rheumatol.

[30] Driscoll T, Jacklyn G, Orchard J, Passmore E, Vos T (2014). The global burden of occupationally related low back pain: estimates from the Global Burden of Disease 2010 study. Ann Rheum Dis.

[31] Strickland FM, Patel D, Khanna D, Somers E, Robida AM (2016). Characterisation of an epigenetically altered CD4(+) CD28(+) Kir(+) T cell subset in autoimmune rheumatic diseases by multiparameter flow cytometry. Lupus Sci Med.

[32] Gorelik GJ, Yarlagadda S, Patel DR, Richardson BC (2012). Protein kinase Cdelta oxidation contributes to ERK inactivation in lupus T cells. Arthritis Rheum.

[33] Sanchez-Ortiz E, Hahm BK, Armstrong DL, Rossie S (2009). Protein phosphatase 5 protects neurons against amyloid-beta toxicity. J Neurochem.

[34] Haslbeck V, Eckl JM, Drazic A, Rutz DA, Lorenz OR (2015). The activity of protein phosphatase 5 towards native clients is modulated by the middle- and C-terminal domains of Hsp90. Sci Rep.

[35] Ripley BJ, Rahman MA, Isenberg DA, Latchman DS (2005). Elevated expression of the Brn-3a and Brn-3b transcription factors in systemic lupus erythematosus correlates with antibodies to Brn-3 and overexpression of Hsp90. Arthritis Rheum.

[36] Strickland FM, Li Y, Johnson K, Sun Z, Richardson BC (2015). CD4(+) T cells epigenetically modified by oxidative stress cause lupus-like autoimmunity in mice. J Autoimmun.

[37] Ghobrial IM, McCormick DJ, Kaufmann SH, Leontovich AA, Loegering DA (2005). Proteomic analysis of mantle-cell lymphoma by protein microarray. Blood.

[38] Weyand CM, Brandes JC, Schmidt D, Fulbright JW, Goronzy JJ (1998). Functional properties of CD4+ CD28− T cells in the aging immune system. Mech Ageing Dev.

[39] Weyand CM, Fulbright JW, Goronzy JJ (2003). Immunosenescence, autoimmunity, and rheumatoid arthritis. Exp Gerontol.

[40] Liuzzo G, Biasucci LM, Trotta G, Brugaletta S, Pinnelli M (2007). Unusual CD4+CD28null T lymphocytes and recurrence of acute coronary events. J Am Coll Cardiol.

[41] Liuzzo G, Goronzy JJ, Yang H, Kopecky SL, Holmes DR (2000). Monoclonal T-cell proliferation and plaque instability in acute coronary syndromes. Circulation.

[42] Liuzzo G, Kopecky SL, Frye RL, O’Fallon WM, Maseri A (1999). Perturbation of the T-cell repertoire in patients with unstable angina. Circulation.

[43] Gorelik G, Sawalha AH, Richardson BC (2010). Lack of PKC delta kinase activity in T cells induces a lupus-like disease. Lupus.

[44] Gorelik G, Richardson B (2012). Oxidative Damage of PKCd in Systemic Lupus Erythematosus. Arthritis Rheum.

[45] Skaggs BJ, Hahn BH, McMahon M (2012). Accelerated atherosclerosis in patients with SLE--mechanisms and management. Nat Rev Rheumatol.

